# Supplementary material for: The GEM-GECO Calcium Indicator Is Useable in Ogataea parapolymorpha Yeast, but Aggravates Effects of Increased Cytosolic Calcium Levels
Source: Int J Mol Sci. 2022 Sep 2;23(17):10004. doi: 10.3390/ijms231710004 (PMC9456557; doi:10.3390/ijms231710004)
Supplement: Supplementary file 1 [file ijms-23-10004-s001.zip › ijms-1841395-supplementary.pdf]

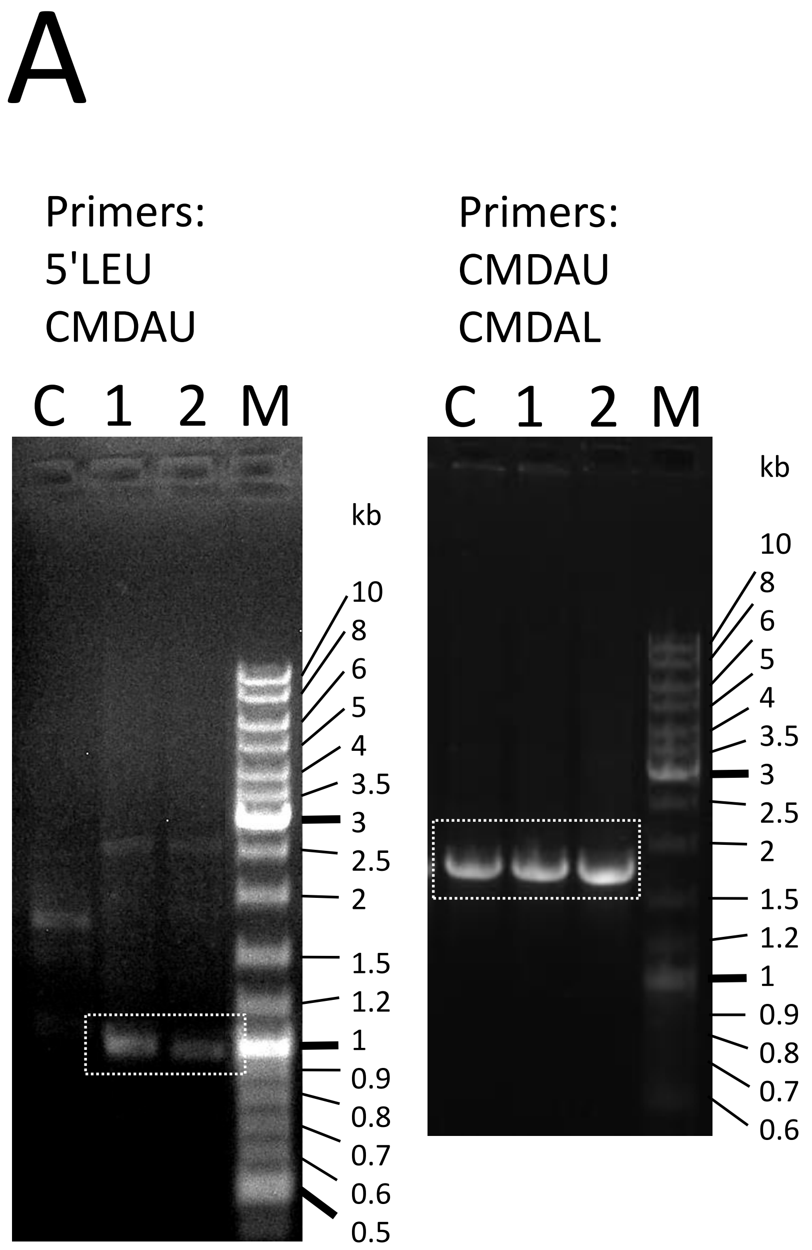

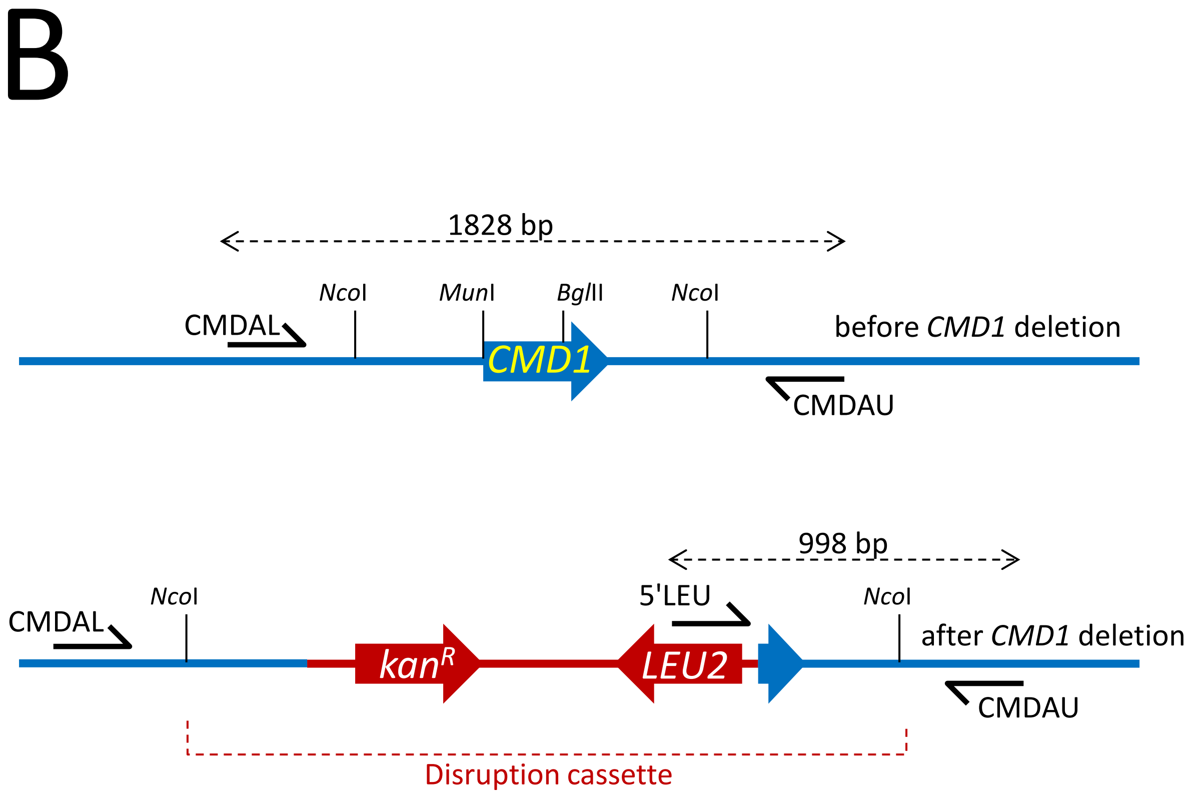

Figure S1. PCR analysis of *O. parapolymorpha* transformants obtained with the pKAM914-derived *CMD1* disruption cassette. A, ethidium bromide stained gels after electrophoresis of PCR products obtained using 5'LEU-CMDAU and CMDAU-CMDAL primers pairs. 5'-3' Sequences of 5'LEU, CMDAU and CMDAL primers are ACCAGGGAGAAGCACAATG, CAACCCGGCCATTCTCGAC and CCACCTCAAGAACCCAACCA, respectively. C , untransformed control strain DNA; 1 and 2, DNA of transformants revealed as *CMD1* disruptants in preliminary screening; M, GeneRuler marker (Thermo). Specific fragments are highlighted by dashed boxes. B, scheme of primer positions in wild-type and disrupted *CMD1* chromosomal loci. Blue lines and arrows, sequences of the *CMD1* genomic locus. Red lines and arrows, sequence of the vector backbone of the pKAM914-derived *CMD1* disruption cassette.

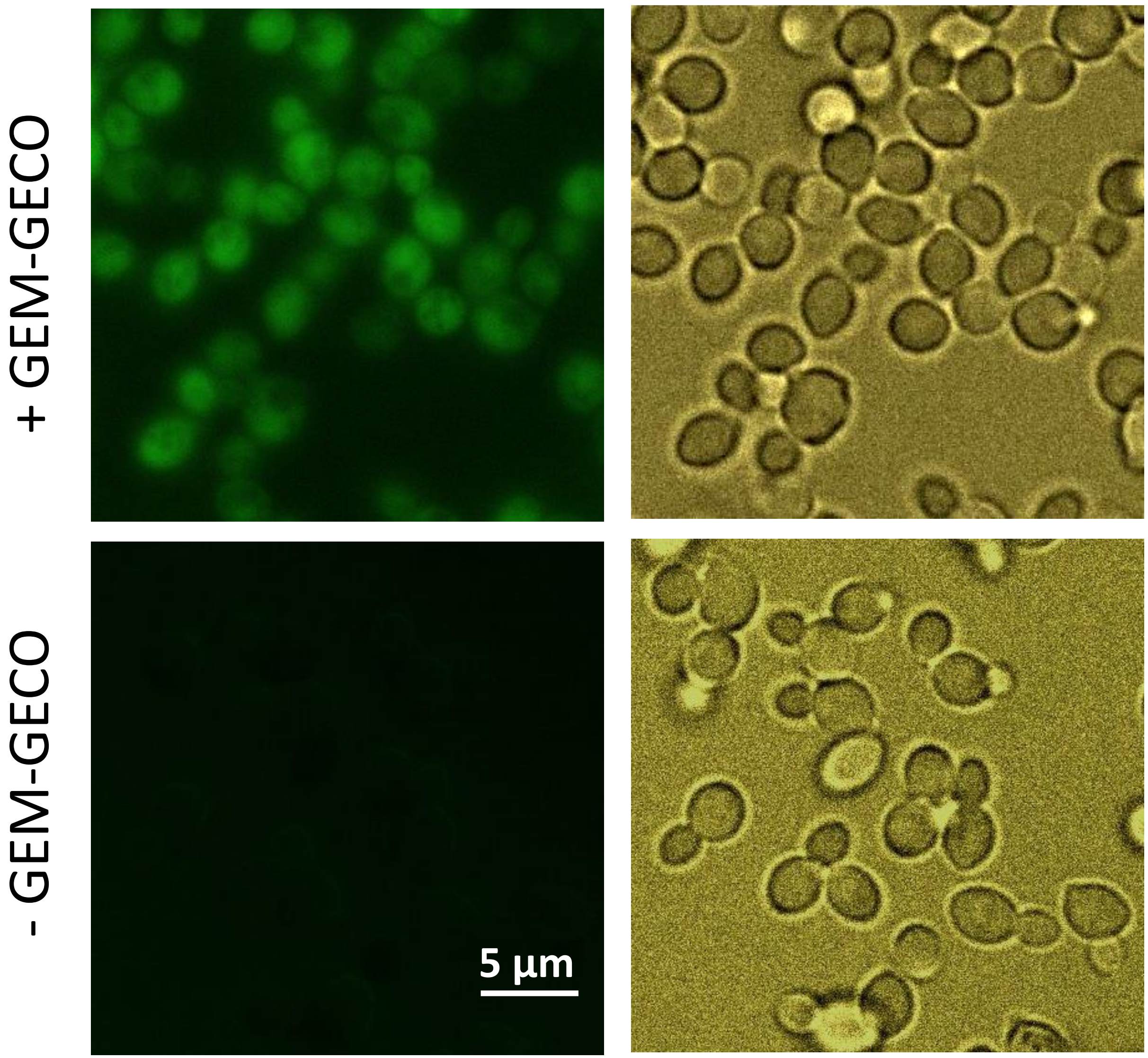

Figure S2. Microphotographs of cells of the *O. parapolymorpha* DL1-L (- GEM-GECO) and DL5 (+ GEM-GECO) strains. Left panel – fluorescence, Ex:395 nm, Em: 510/20 nm; right panel – diascopic image.

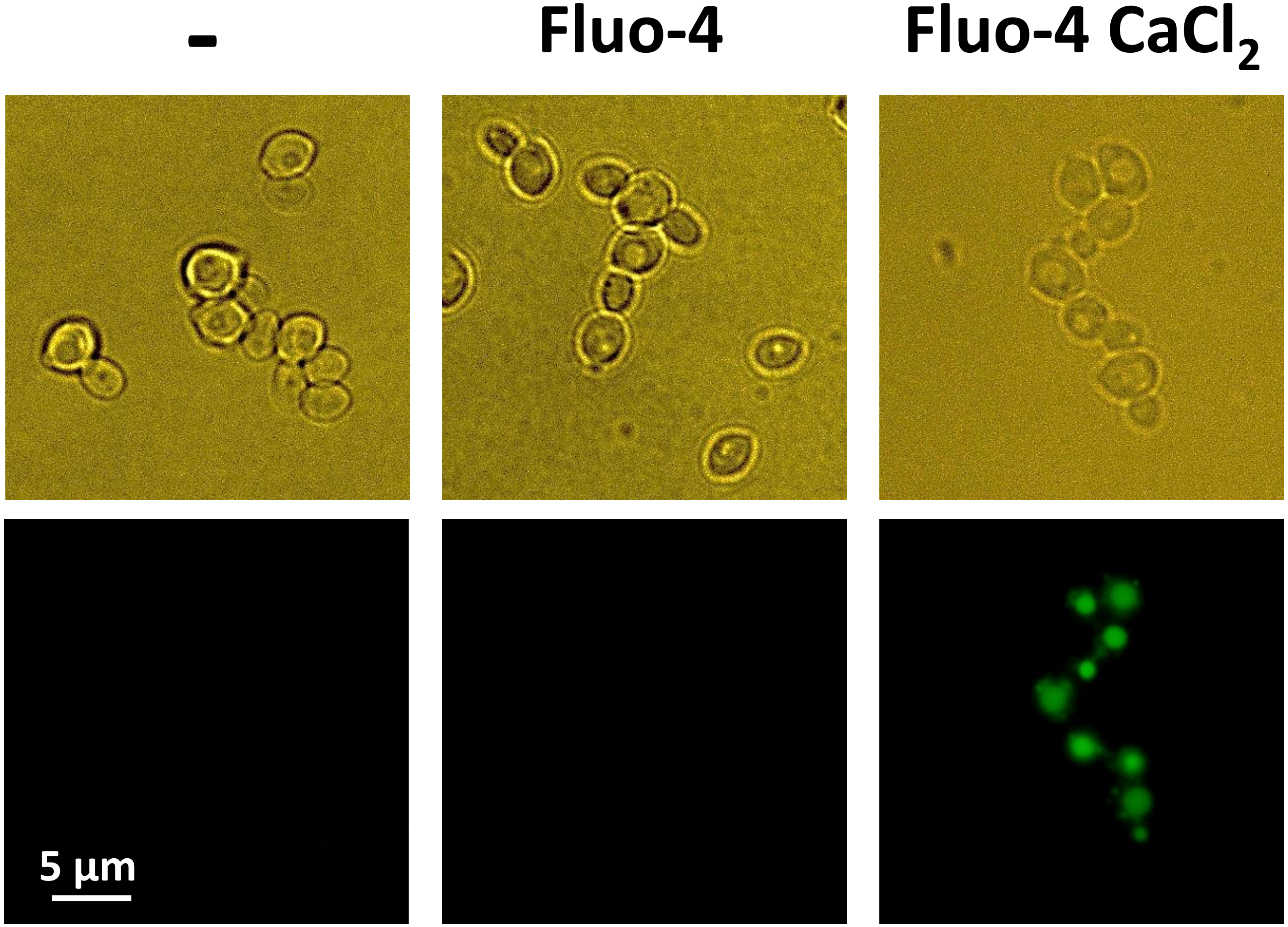

Figure S3. Staining the *O. parapolymorpha* DL1-L cells with Fluo-4-AM. Upper panel, diascopic image; Lower panel, fluorescence, Ex:488 nm, Em: 510/20 nm; "-", unstained cells; "Fluo-4", Fluo-4-loaded cells incubated in regular YPD; "Fluo-4 $CaCl_2$" , Fluo-4-loaded cells incubated in YPD supplemented with 100 mM $CaCl_2$.

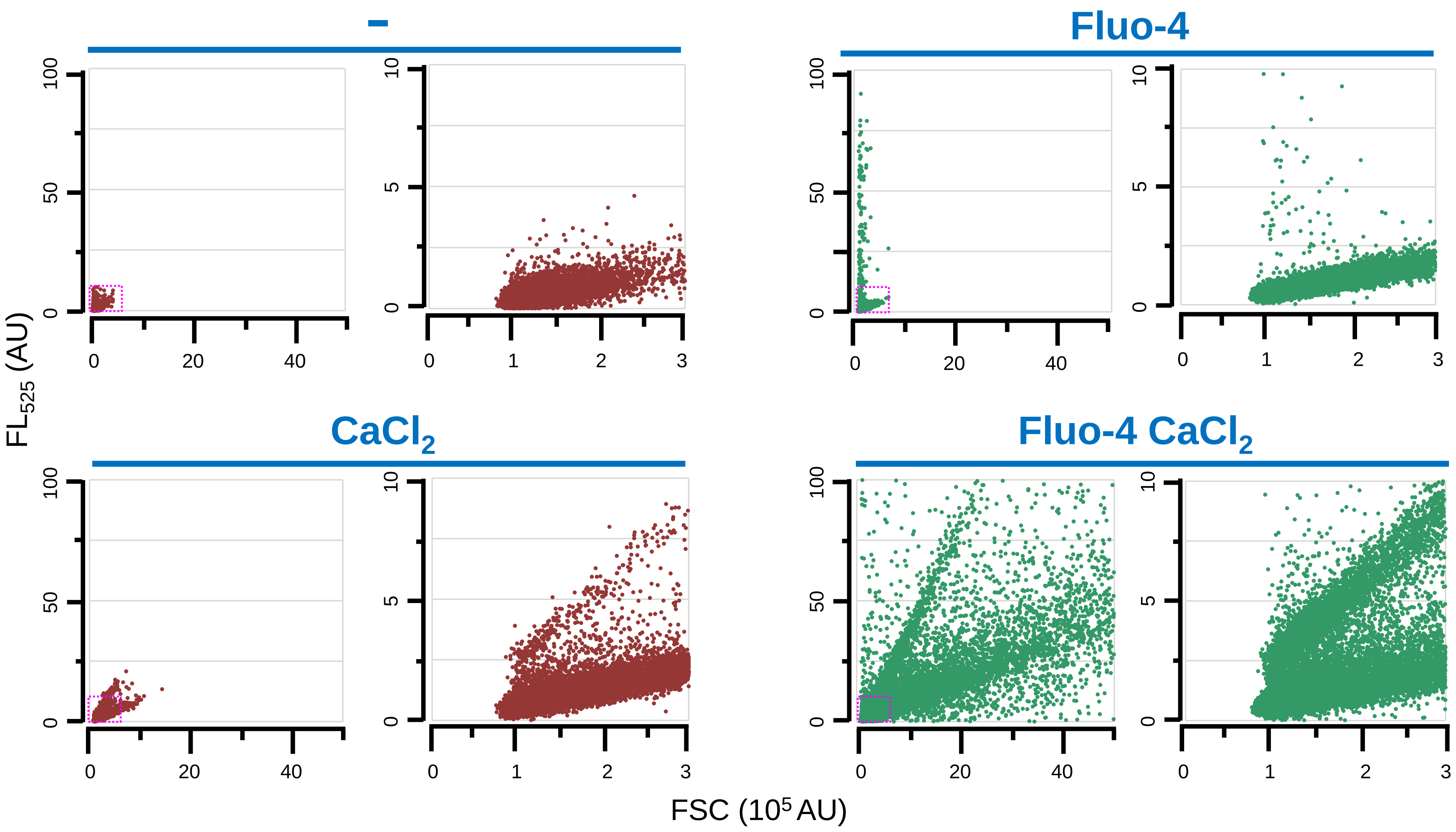

Figure S4. Pairs of scatter plot diagrams of distribution of *O. parapolymorpha* DL1-L cells according to their 488 nm excited fluorescence at 525 nm (FL_{525}) and forward scattering (FSC) in YPD culture before (-) and after (Fluo-4) staining with the Fluo-4-AM dye. To test effects of external Ca^{2+} on the Fluo-4 fluorescence, 100 mM $CaCl_2$ was added to unstained ($CaCl_2$) or Fluo-4-AM-stained (Fluo-4 $CaCl_2$) culture and incubated for 5 min prior to flow cytometric analysis. Right panels show zoomed-in areas marked by dotted boxes on the left panels.

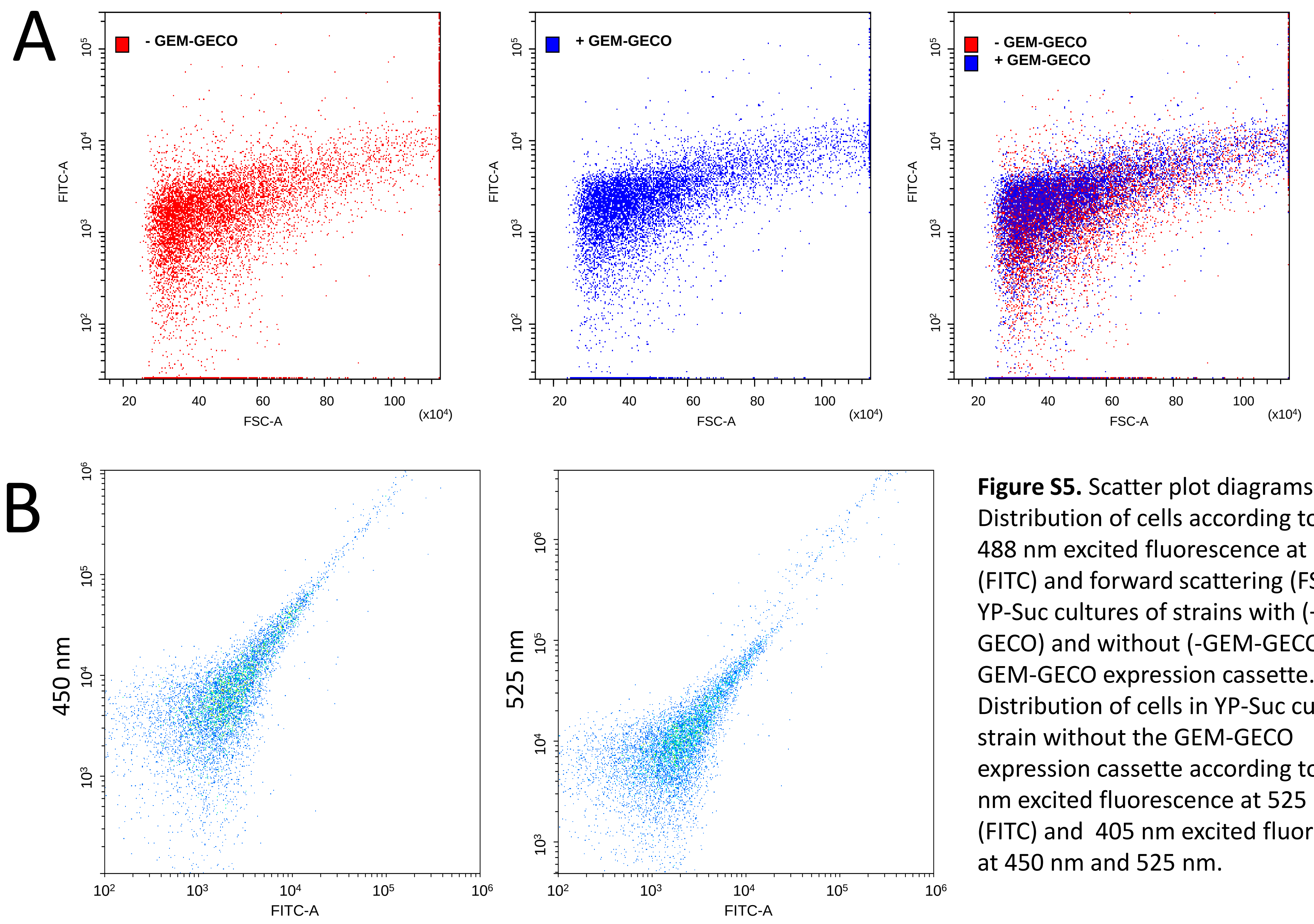

Figure S5. Scatter plot diagrams. A, Distribution of cells according to their 488 nm excited fluorescence at 525 nm (FITC) and forward scattering (FSC-A) in YP-Suc cultures of strains with (+GEM-GECO) and without (-GEM-GECO) the GEM-GECO expression cassette. B, Distribution of cells in YP-Suc culture of strain without the GEM-GECO expression cassette according to 488 nm excited fluorescence at 525 nm (FITC) and 405 nm excited fluorescence at 450 nm and 525 nm.

Table S1. Phenotypes of randomly selected haploid segregants of the diploid strain containing the *CMD1* disruption allele (*ade2/ade2 URA3/ura3::ADE2 MOX/mox::uPA* P_{MAL1}*:GEM-GECO/- CMD1/cmd1::LEU2*)

Phenotype	# of clones
Mut^- Ade^- Leu^- Ura^+ GEM-$GECO^+$	9
Mut^- Ade^- Leu^- Ura^+ GEM-$GECO^-$	10
Mut^+ Ade^- Leu^- Ura^+ GEM-$GECO^+$	11
Mut^+ Ade^- Leu^- Ura^+ GEM-$GECO^-$	9
Mut^+ Ade^+ Leu^- Ura^+ GEM-$GECO^-$	2
Mut^+ Ade^+ Leu^- Ura^- GEM-$GECO^+$	3
Mut^+ Ade^+ Leu^- Ura^- GEM-$GECO^-$	4
Mut^- Ade^+ Leu^- Ura^- GEM-$GECO^+$	3
Mut^- Ade^+ Leu^- Ura^- GEM-$GECO^-$	5

Table S2. Ratio of clones with specified phenotypes among randomly selected haploid segregants of the diploid strain containing the *CMD1* disruption allele (*ade2/ade2 URA3/ura3::ADE2 MOX/mox::uPA* P_{MAL1}*:GEM-GECO/- CMD1/cmd1::LEU2*)

Phenotypes	Ratio
Mut^+:Mut^-	29:27
Ura^+ Ade^-:Ura^- Ade^+	39:15
Leu^+:Leu^-	0:56
GEM-$GECO^+$:GEM-$GECO^-$	26:30
